# A retrospective cohort study on association of age and physician decision making for or against rapid sequence intubation in unconscious patients

**DOI:** 10.1038/s41598-022-06787-3

**Published:** 2022-02-28

**Authors:** Michael Eichlseder, Michael Eichinger, Barbara Hallmann, Gabriel Honnef, Philipp Metnitz, Gerhard Prause, Philipp Zoidl, Paul Zajic

**Affiliations:** Division of General Anaesthesiology, Emergency- and Intensive Care Medicine, Medical University of Graz, Auenbruggerplatz 5, 8036 Graz, Austria

**Keywords:** Risk factors, Geriatrics, Health services

## Abstract

In unconscious individuals, rapid sequence intubation (RSI) may be necessary for cardiopulmonary stabilisation and avoidance of secondary damage. Opinions on such invasive procedures in people of older age vary. We thus sought to evaluate a possible association between the probability of receiving prehospital RSI in unconsciousness and increasing age. We conducted a retrospective study in all missions (traumatic and non-traumatic) of the prehospital emergency physician response unit in Graz between January 1st, 2010 and December 31st, 2019, which we searched for Glasgow Coma Scale (GCS) below 9. Cardiac arrests were excluded. We performed multivariable regression analysis for RSI with age, GCS, independent living, and suspected cause as independent variables. Of the 769 finally included patients, 256 (33%) received RSI, whereas 513 (67%) did not. Unadjusted rates of RSI were significantly lower in older patients (aged 85 years and older) compared to the reference group aged 50–64 years (13% vs. 51%, p < 0.001). In multivariable regression analysis, patients aged 85 years and older were also significantly less likely to receive RSI [OR (95% CI) 0.76 (0.69–0.84)]. We conclude that advanced age, especially 85 years or older, is associated with significantly lower odds of receiving prehospital RSI in cases of unconsciousness.

## Introduction

Loss of consciousness is a common reason for prehospital emergency service activations^1^. There is a plethora of potential causes for sudden loss of consciousness; the most prevalent are cerebrovascular events, seizures, intoxication, traumatic brain injury, respiratory failure, and metabolic disorders^2,3^. Due to this heterogeneity of underlying conditions, definitive therapy and prognosis vary considerably. However, regardless of the cause, the cornerstones of emergency care for unconscious patients are manoeuvres to secure the airway, maintain adequate oxygenation, enable sufficient ventilation, and establish stable haemodynamic conditions in order to avoid secondary organ damage^4,5^.

In the Austrian prehospital emergency medical system, physicians are dispatched to calls perceived as life-threatening, such as severe trauma, unconsciousness, or cardiac arrest. Advanced medical interventions, such as induction of anaesthesia and endotracheal intubation (ETI), are already performed at the scene of the event. To minimise the risk of aspiration, ETI should be performed as a rapid sequence intubation (RSI)^6^. All three components of anaesthesia—hypnosis, analgesia, and neuro-muscular blockade—are induced simultaneously and as fast as possible while mask ventilation is avoided whenever feasible^6^.

Prehospital RSI is an invasive intervention with significant associated risk; the decision to perform the procedure must therefore be well considered. Hypoxaemia, hypotension, and arrhythmia are common during RSI outside the operating theatre, the view of the larynx is often not ideal. First pass success in a comparable system was only 75%^7,8^. In addition to patient-related factors, such as the cause and severity of the impairment of consciousness, cardiorespiratory impairment, and previous illnesses, process-related elements, such as the experience of the physician performing the procedure, and transport time to the next hospital, should also be taken into account in order to make the best possible decision for patients^6^.

Due to ongoing shifts in demographics, people of old age make up an ever-growing proportion of today’s societies. Despite the fact that first pass success in people of old age has been shown to be higher than in younger individuals^9^, outcome quality and life years to be generated by medical interventions in acute illness or injury may be limited. Opinions on life-sustaining invasive procedures, such as mechanical ventilation and intensive care admission, in people of older age range from futile to worthwhile^10–12^. This may be especially true in frail patients^13^, since frailty represents a chronic state of reduced physiological reserve and an increased vulnerability to exogenic stressors due to an age associated functional decline^14^. Patient age could therefore be another factor in the decision-making process in the prehospital phase.

### Aim of this study

The aim of this study was to evaluate whether age is a factor significantly associated with the decision to perform rapid sequence intubation in unconscious patients in the prehospital setting, irrespective of other confounding factors such as the suspected cause of the current condition and care dependency.

## Methods

### Study design and data source

This study was a single-centre, retrospective review of prospectively collected routine data. Data were retrieved from the database of the prehospital emergency physician response system located at a University Medical Centre. This response system is staffed with a prehospital emergency physician, specialised in either anaesthesia, internal medicine or surgery, and a paramedic. It is dispatched around 2000 times a year to take care of approximately 200,000 individuals in the eastern part of the city and its suburban area.

Routine medical documentation based on the minimal dataset for emergencies (MIND) laid out by the German Interdisciplinary Society of Intensive Care and Emergency Medicine (DIVI), which is electronically collected and stored (MEDEA, iLogs, Klagenfurt, Austria), was retrieved. Routine documentation includes, but is not limited to, Glasgow Coma Score (GCS), National Advisory Committee for Aeronautics Score (NACA), and suspected diagnosis^15,16^.

### Ethical approval and need for consent

Ethical approval was sought and granted by the ethics committee of the Medical University of Graz (IRB00002556), decision number 30–373 ex 17/18 before data retrieval and study conduction. The need for informed consent was waived as data were retrieved and analysed retrospectively and in a pseudonymised fashion.

All used methods and performed analyses were carried out in accordance with relevant guidelines (STROBE statement) and regulations (especially the European General Data Protection Regulation).

### Patient selection, data extraction and preparation

Data of missions between January 1st, 2010 and December 31st, 2019 were extracted. Cases in which physicians treated unconscious patients (defined as GCS below 9 upon arrival at the scene until the end of the mission) were selected. Patients with cardiac arrest, interhospital transfers, blank datasets (e.g., due to technical difficulties of the documentation system), patients with missing age data, and patients who were already treated by another physician on scene were excluded.

The following variables for analyses were derived: age, GCS, and anonymised physician identifier. If the age was not exactly known or documented, the treating prehospital physician’s estimate was used. These data were checked for plausibility both electronically and manually. Using the United Nations age categories adapted to create comparable group sizes, the patient cohort was divided into the following age groups: under 18 years, 18–29 years, 30–49 years, 50–64 years, 65–75 years, 75–84 years, and 85 years and older.

Further data retrieved were: conduction of RSI, suspected cause of unconsciousness, and care dependency. RSI was considered conducted if anaesthetic drugs were administered and an advanced airway (either an endotracheal tube, a supraglottic airway device, or a surgical airway) was placed. Each case was allocated to one of seven predefined causes (trauma, medical, toxicology, seizure, cerebrovascular, other, and unknown) of unconsciousness. Documented patient history, clinical examination, and prehospital diagnosis were used as a basis, which were evaluated by two researchers independently and discussed in the team in cases of disagreement. Consensus was reached in all cases. Care dependency was considered if explicitly mentioned in free text documentation or if the patient was living in a nursing home. The anonymous dataset used for this study can be found in the electronic supplementary materials attached to this manuscript.

### Statistical analysis

Factors possibly influencing the decision to perform RSI were presented as median and interquartile ranges (IQR) or number (*n*) and percentages (%), as appropriate. Between-group comparisons were performed using a Kruskal–Wallis test or Chi-square test, as appropriate. Bonferroni correction was used to adjust for multiple testing, *p*-values below 0.05 were considered significant.

A multivariable mixed linear regression model with RSI as the dependent variable and the aforementioned possible factors as independent variables was constructed; these were: age in categories (under 18 years, 18–29 years, 30–49 years, 50–64 years, 65–74 years, 75–84 years, and 85 years and older; 50–64 years, being the median age group, was chosen as the reference group), independent living, GCS, and suspected cause of unconsciousness. The model was further adjusted for anonymised identifiers of physicians making the decision as random effects.

For sensitivity analysis, the model was repeated in patients with non-traumatic suspected causes for unconsciousness only. Receiver Operating Characteristics (ROC) analyses were performed to assess predictive capabilities of these models.

All analyses were performed using IBPM SPSS 26 (IBM Corp, Armonk, NY, USA).

### IRB identifier and decision

Ethics committee of the Medical University of Graz (IRB00002556), decision number 30–373 ex 17/18.

## Results

**Figure 1:**
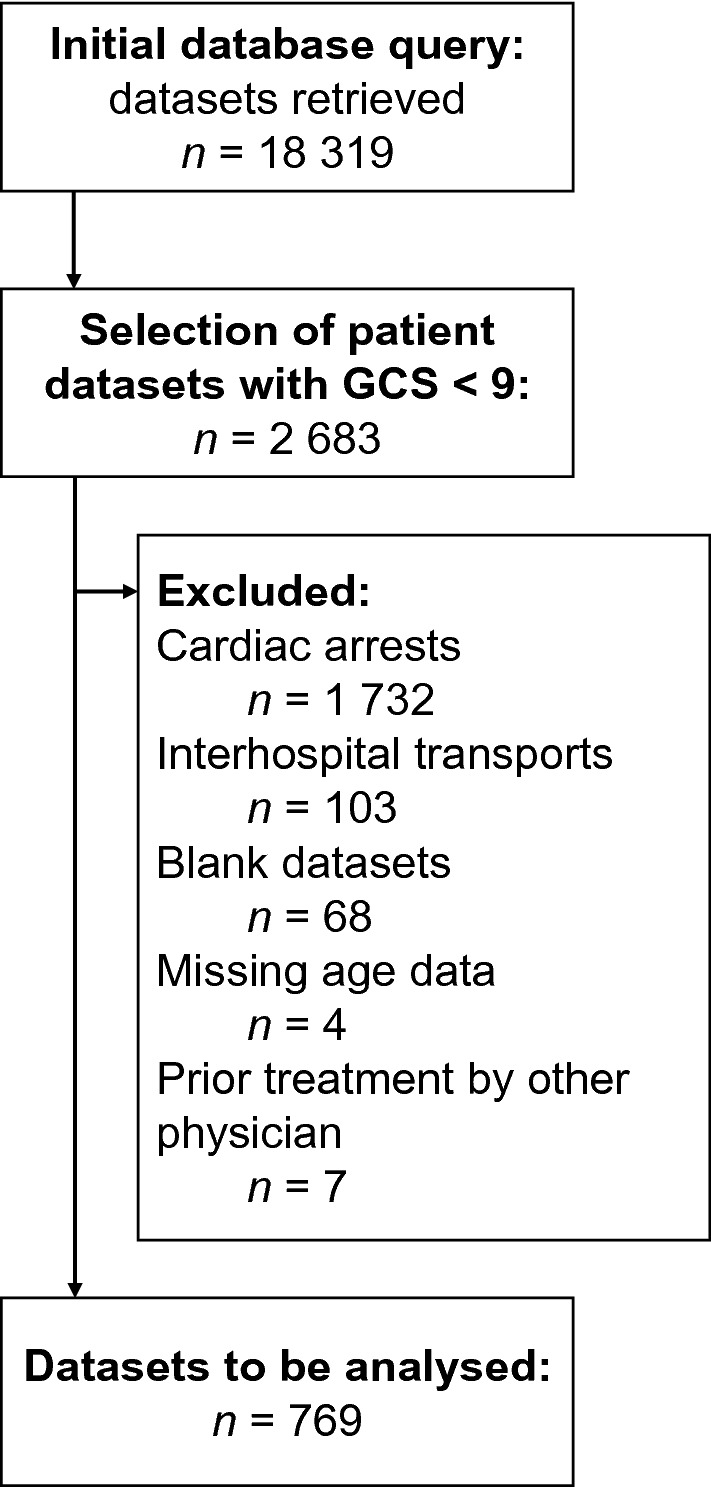
Study flow chart.

The initial database query and application of inclusion and exclusion criteria yielded data on 769 patients who presented with unconsciousness. The selection process is depicted in Fig. 1. In this cohort, 256 (33%) patients received RSI whereas 513 (67%) did not. 65 physicians, conducting a median of 2 (IQR 1–6) RSIs in the observation period, made the decisions in question.

### Unadjusted comparative analysis

Patients who received RSI were significantly younger [median age 61 (41–76) vs. 72 (36–86) years, p < 0.001], had significantly lower Glasgow Coma Scores [4 (3–6) vs. 6 (4–7), p < 0.001], and were significantly less often care dependent [13 (9%) vs. 137 (91%), p < 0.001] (Table 1) as compared to patients who were not intubated.

**Table 1:** Patient characteristics in the overall cohort and comparisons between those patients who received prehospital rapid sequence intubation and those who did not.

	Overall cohort	Prehospital RSI		*p*
		No	Yes	
*n* of patients	769	513 (67%)	256 (33%)	
Age [years] (median, IQR)	67 (39–84)	72 (36–86)	61 (41–76)	< 0.001
**Age category (n, %)**				< 0.001
< 18 years	66	50 (76%)	16 (24%)	
18–29 years	72	52 (72%)	20 (28%)	
30–49 years	108	63 (58%)	45 (42%)	
50–64 years	122	60 (49%)	62 (51%)	
65–74 years	94	51 (54%)	43 (46%)	
75–84 years	126	80 (64%)	46 (36%)	
≥ 85 years	181	157 (87%)	24 (13%)	
Care dependency (n, %)	150	137 (91%)	13 (9%)	< 0.001
GCS (median, IQR)	6 (3–7)	6 (4–7)	4 (3–6)	< 0.001
**Suspected cause (n, %)**				< 0.001
Trauma	78	8 (10%)	70 (90%)	
Medical	115	92 (80%)	23 (20%)	
Cerebrovascular	165	95 (58%)	70 (42%)	
Seizure	157	136 (87%)	21 (13%)	
Intoxication	89	61 (69%)	28 (31%)	
Uncertain	122	84 (69%)	38 (31%)	
Other	43	37 (86%)	6 (14%)	

*GCS* Glasgow Coma Score, *IQR* inter-quartile range, *RSI* rapid sequence intubation.

**Figure 2:**
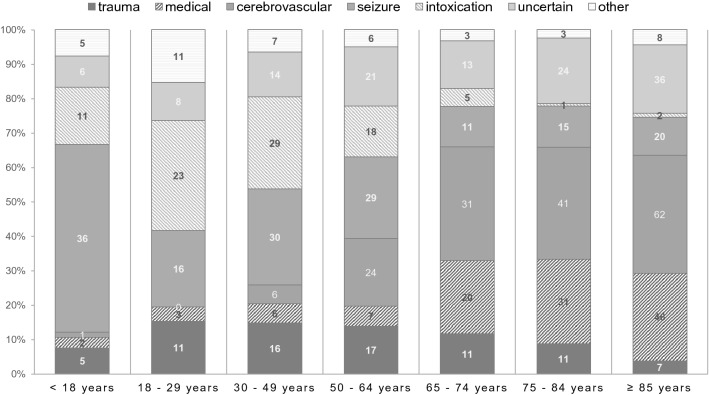
Suspected causes of unconsciousness in different age groups. Bars indicate distribution of proportions within the respective age groups, numbers represent absolutes.

There were significant differences in patient characteristics between the different age groups. Care dependency was progressively more common in individuals of older age [19/94 (20%) in 65- to 74-year-olds, 38/126 (30%) in 75- to 84-year-olds and 69/122 (38%)] in those aged 85 or above, p < 0.001). The suspected reason for unconsciousness also varied greatly with age; while in individuals below 18 years of age, seizures were the most commonly suspected cause [36/66 (55%)], those of older age presented mainly with signs and symptoms of cerebrovascular events [41/216 (33%) and 62/122 (34%) for 75–84 years and ≥ 85 years, respectively] and medical conditions [31/216 (25%) and 46/122 (25%) for 75–84 years and ≥ 85 years, respectively] (Table 2, Fig. 2).

**Table 2:** Patient characteristics in the overall cohort and comparisons between age groups.

	Overall cohort	Age group							*p*
		< 18 years	18–29 years	30–49 years	50–64 years	65–74 years	75–84 years	≥ 85 years	
*n* of patients	769	66	72	108	122	94	126	122	
Age [years] (median, IQR)	67 (39–84)	8 (2–14)	25 (21–27)	39 (33–45)	57 (54–62)	71 (68–73)	81 (78–83)	89 (87–92)	n/a
Care dependency (n, %)	150 (20%)	5 (8%)	4 (6%)	4 (4%)	11 (9%)	19 (20%)	38 (30%)	69 (38%)	< 0.001
GCS (median, IQR)	6 (3–7)	5 (3–7)	6 (3–8)	6 (3–7)	5 (3–7)	6 (4–7)	6 (3–7)	6 (4–7)	0.23
**Suspected cause (n, %)**									< 0.001
Trauma	78 (10%)	5 (8%)	11 (15%)	16 (15%)	17 (14%)	11 (12%)	11 (9%)	7 (4%)	
Medical	115 (15%)	2 (3%)	3 (4%)	6 (6%)	7 (6%)	20 (21%)	31 (25%)	46 (25%)	
Cerebrovascular	165 (22%)	1 (2%)	0 (0%)	6 (6%)	24 (20%)	31 (33%)	41 (33%)	62 (34%)	
Seizure	157 (20%)	36 (55%)	16 (22%)	30 (28%)	29 (24%)	11 (12%)	15 (12%)	20 (11%)	
Intoxication	89 (12%)	11 (17%)	23 (32%)	29 (27%)	18 (15%)	5 (5%)	1 (1%)	2 (1%)	
Uncertain	122 (16%)	6 (9%)	8 (11%)	14 (13%)	21 (17%)	13 (14%)	24 (29%)	36 (20%)	
Other	43 (6%)	5 (8%)	11 (15%)	7 (7%)	6 (5%)	3 (3%)	3 (2%)	8 (4%)	
Prehospital RSI (n, %)	256 (33%)	16 (24%)	20 (28%)	45 (42%)	62 (51%)	43 (46%)	46 (37%)	24 (13%)	< 0.001

*GCS* Glasgow Coma Score, *IQR* inter-quartile range, *n/a* not applicable, *RSI* rapid sequence intubation.

Rate of RSI was also significantly different between age groups. The highest proportion of unconscious patients who received prehospital RSI was found in the age group of 50 to 64 years [62/122 (51%)]. Patients of higher age were progressively less likely to receive prehospital RSI [43/94 (46%) for 65- to 74-year-old patients, 46/126 (37%) for 75- to 84-year-old patients, and 24/122 (13%) for those aged 85 years or above, p < 0.001] (Table 2). Similarly, patients of younger age also received RSI less often; the lowest percentage was found in patients below 18 years [16/66 (24%)].

### Adjusted analysis

In a multivariable regression analysis, age 85 years or above was associated with lower odds of prehospital RSI than the reference group of age between 50 and 64 years [OR (95% CI) 0.76 (0.69–0.84)]. No significant differences from the reference group were found for age 30 to 49 years, 65 to 74 years, and 75 to 84 years. Of note, younger age was also associated with lower odds of prehospital RSI [OR 0.88 (0.79–0.99) for < 18 years, and 0.84 (0.76–0.95) for 18 to 29 years, respectively] (Table 3).

**Table 3:** Multivariable mixed linear regression model for prehospital RSI as the dependent variable.

	OR	95% CI	
**Age category**			
< 18 years	0.88	0.79	0.99
18–29 years	0.84	0.76	0.95
30–49 years	0.95	0.86	1.05
50–64 years	1.00		
65–74 years	0.98	0.88	1.09
75–84 years	0.91	0.83	1.01
≥ 85 years	0.76	0.69	0.84
Care dependency	0.83	0.77	0.90
**Suspected cause**			
Trauma	1.00		
Medical	0.60	0.53	0.67
Cerebrovascular	0.68	0.61	0.75
Seizure	0.51	0.46	0.57
Intoxication	0.55	0.49	0.62
Uncertain	0.52	0.45	0.60
Other	0.61	0.55	0.68

Model also adjusted for physician identifier as random effect. AUROC = 0.85.

*CI* confidence interval, *OR* odds ratio.

Care dependency was also associated with lower odds of RSI [OR (95% CI) 0.83 (0.77–0.90)]. All other suspected causes for unconsciousness were associated with drastically lower odds for prehospital RSI than the reference category of trauma (Table 3). The chosen variables allowed for good prediction, AUROC for this model was 0.85.

Excluding cases with unconsciousness due to suspected trauma (n = 78) demonstrated stability of the effect over the remaining groups. Age 85 years and above was associated with lower odds of prehospital RSI [OR (95% CI) 0.75 (0.68–0.83)] (Table 4); AUROC for this model was 0.81.

**Table 4:** Multivariable mixed linear regression model for prehospital RSI as the dependent variable in patients with suspected non-traumatic causes of unconsciousness.

	OR	95% CI	
**Age category**			
< 18 years	0.85	0.76	0.97
18–29 years	0.82	0.73	0.93
30–49 years	0.94	0.84	1.05
50–64 years	1.00		
65–74 years	0.98	0.87	1.10
75–84 years	0.91	0.82	1.01
≥ 85 years	0.75	0.68	0.83
Care dependency	0.83	0.77	0.90
**Suspected cause**			
Medical	0.98	0.89	1.08
Cerebrovascular	1.11	1.02	1.22
Seizure	0.84	0.76	0.92
Intoxication	0.92	0.82	1.03
Uncertain	0.87	0.76	1.00
Other	1.00		

Model also adjusted for physician identifier as random effect. AUROC = 0.81.

*CI* confidence interval, *OR* odds ratio.

## Discussion

This study investigated whether old age is associated with the decision to perform prehospital rapid sequence intubation in unconscious patients. In a multivariable model it was demonstrated that individuals of older age—i.e., 85 years or above—are less likely to receive an RSI in case of unconsciousness compared to other age groups irrespective of other potentially influential factors.

This finding is similar to those of a previous study conducted in a similarly structured prehospital system in 21,922 patients in Switzerland. The authors concluded that advanced airway management in patients NACA 4 or higher is significantly less likely in patients 89 years and older compared to younger patients^17^.

In a retrospective study conducted in a cohort of 1681 patients receiving an RSI in an emergency department, patients aged 80 years and above only accounted for 6% at that unit. Despite this high degree of selection, only 20% of the patients aged 80 years and older who received an RSI in that study survived to hospital discharge^18^. A possible explanation for this finding is that clinicians consider these invasive emergency procedures and subsequent intensive care treatment to be futile in octogenarians and nonagenarians and therefore refrain from performing such interventions.

More than three quarters of physicians participating in the institution’s physician response system also work in intensive care. Knowledge of common further course of treatment of these patients and their high mortality could have a pronounced influence on decision-making.

In other studies, age alone was of stronger influence than patients’ functional status when a decision had to be made in the emergency department whether to engage in intensive care or not^19^. In severely ill patients, older age is associated with higher rates of withholding life-sustaining treatments despite same life expectancy. This was shown in a prospective cohort study with 9105 patients suffering from an illness with an average 6-month mortality of 50% where, even after adjustment for prognosis and disease, ventilatory support, major surgery and dialysis was significantly less likely in patients 80 years and older^20^. These data do not include comorbidities, but it is likely that age might be an indirect indicator of frailty or illness. However, taking a detailed and all-encompassing patient history in prehospital emergency care is often impossible, while decisions have to be made within minutes. Consequently, age is often the first, and sometimes the only patient characteristic available.

Need for nursing care was found to be another factor associated with significantly lower odds of RSI. Patients living in nursing homes are often frail and more likely to have multiple co-morbidities^21^. Living in a nursing home is associated with decreased odds of undergoing invasive procedures such as intubation and mechanical ventilation in the last 6 months of life^22^. 90-day mortality of nursing home residents after admission to an intensive care unit has been reported at 50% in a retrospective study and the benefit of invasive interventions has therefore been questioned by some authors^23,24^. Again, awareness of these effects could at least be part of the reason for the lower rate of RSI in this cohort.

Patients with suspected traumatic origin of unconsciousness receive RSI almost universally; odds for RSI are therefore vastly higher compared to non-traumatic causes. This can, at least partly, be explained by the GCS itself, as this score is validated primarily for patients with traumatic brain injury^25–27^. This is also reflected in current guidelines. While prehospital intubation and ventilation are strongly encouraged in trauma patients with a GCS of less than 9, recommendations in non-traumatic patients are rather vague. Non-traumatic patients should receive RSI “in case of unconsciousness in combination with a high risk of aspiration”. Cut-off values for GCS are not defined^6,28^. RSI rates are 90% in traumatic and 23% in non-traumatic unconsciousness patients^29,30^. Furthermore, a shift towards cerebrovascular and medical origin of unconsciousness was notable in older patients. Intracranial haemorrhages are associated with very high rates of mortality or severe impairment in this age group^31^. Respiratory failure, the most common medical cause for unconsciousness in older individuals, often marks the final stage of morbidity and is thus associated with high mortality rates^32^. This shift seems to be relevant with regards to the decision-making processes and the suspected cause of unconsciousness.

Findings from our study suggest, that suspected cause of altered mental status, care dependency, and age are all associated with physicians’ decision for or against performing prehospital RSI in unconscious individuals. As impactful decision making in stressful and information-limited environments, such as prehospital emergency medicine, is demanding and thus morally and medically difficult, future research may focus on the development and introduction of standardised decision tools to aid treating health care professionals in their decision processes.

### Strengths and limitations

This is a single-centre retrospective study in a physician-based emergency response system in central Europe. Results cannot necessarily be generalised to other systems, regions, and countries. The available data set is restricted to its original contents and certain variables, such as past medical history and comorbidities, which might be relevant for the decision-making process, are not completely available. Furthermore, evaluators were not blinded regarding age and outcome in this study. However, these data represent real-world situations in which health-care professionals have to deal with very limited information on scene in the prehospital setting. Based on this limited information, the decision for or against invasive procedures has to be made (Supplementary Information).

## Conclusion

Individuals aged 85 years or older are significantly less likely to receive prehospital rapid sequence intubation in cases of unconsciousness than individuals in our reference group, aged 50 to 65 years. This is probably mostly due to differences in suspected reasons for unconsciousness and expected chances of recovery rather than due to age itself.

## Supplementary Information

Supplementary Information.

## Data Availability

Anonymous data used for analyses in this study are supplied together with this manuscript.
